# The Latest Research Progress of m^6^A Modification and Its Writers, Erasers, Readers in Infertility: A Review

**DOI:** 10.3389/fcell.2021.681238

**Published:** 2021-09-10

**Authors:** Xuda Liu, Haiying Wang, Bingchen Liu, Zhipeng Qi, Jiashuo Li, Bin Xu, Wei Liu, Zhaofa Xu, Yu Deng

**Affiliations:** Department of Public Health, China Medical University, Shenyang, China

**Keywords:** m^6^A modification, methyltransferase, demethylase, binding protein, gametogenesis, infertility

## Abstract

Eukaryotic messenger mRNAs contain many RNA methyl chemical modifications, in which N^6^-methyladenosine (m^6^A) plays a very important role. The modification process of RNA methylation is a dynamic reversible regulatory process that is mainly catalyzed by “Writer” m^6^A methyltransferase, removed by “Eraser” m^6^A demethylase, and recognized by the m^6^A binding protein, thereby, linking m^6^A modification with other mRNA pathways. At various stages of the life cycle, m^6^A modification plays an extremely important role in regulating mRNA splicing, processing, translation, as well as degradation, and is associated with gametogenesis and fertility for both sexes. Normal gametogenesis is a basic guarantee of fertility. Infertility leads to trauma, affects harmony in the family and seriously affects the quality of life. We review the roles and mechanisms of RNA m^6^A methylation modification in infertility and provide a potential target for infertility treatment, which can be used for drug development.

## Overview of Infertility

**FIGURE 1:**
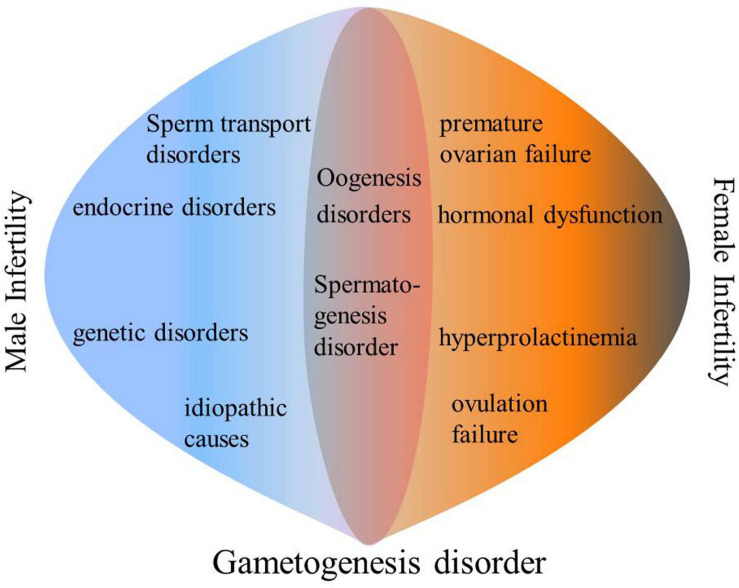
Main causes of infertility.

Infertility is a reproductive dysfunction that is characterized by a succession of sexual events and interruption of new life. Accordingly, an inability to get pregnant after 12 months of unprotected intercourse is broadly defined as infertility (Petraglia et al., 2013; Kayode et al., 2020). Infertility can be divided into female infertility and male sterility. Female infertility is defined as: for women under 35 years of age, they cannot get pregnant after 12 months of unprotected sex, or for women over 35, unprotected intercourse for 6 months cannot lead to pregnancy. The most common infertility factor is ovulation disorder, which accounts for about 25% of all infertility factors (“Practice Committee of the American Society for Reproductive Medicine,Practice Committee of the American Society for Reproductive Medicine., 2013” Recent advances in medically assisted conception. Oogenesis is a complex process that is regulated by many internal and external ovarian factors (Sánchez and Smitz, 2012). Oogenesis disorders can lead to ovulation disorders, which in turn lead to female infertility. According to the World Health Organization (WHO), male infertility refers to a person who cannot cause a pregnancy after at least 12 months of unprotected sexual intercourse (La Vignera et al., 2011). The four main causes of male infertility include: sperm transport disorders, endocrine disorders, genetic disorders and idiopathic causes (Kayode et al., 2020). Among them, idiopathic causes account for 40% of male infertility cases (Louis et al., 2013). Spermatogenesis is a sophisticated developmental process in which haploid sperms are continuously produced by diploid spermatogonia. Frequent cell division and differentiation cause epigenetic modification changes and genetic distortion during chromosomal remodeling that eventually inhibit spermatogenesis, leading to low sperm concentrations, low motor abilities and poor morphologies (Clermont, 1972; de Rooij, 2001; Rajender et al., 2011; Battle, 2013; Griswold, 2016). Therefore, a large number of idiopathic male infertility cases are due to genetic aberrations (Figure 1).

## Gametogenesis

The process through which mature germ cells are formed is called gametogenesis. Gametogenesis is indispensable to the general health and well-being of various species. Overtime, gametes link the various stages of species’ development. Compared to somatic cells, after a series of mitosis and meiosis during the differentiation process, germ cells produce sperms and oocytes. Sexually mature gametes combine during fertilization to produce the all-powerful oosperm (Carreau and Hess, 2010). An important aspect of gametogenesis is meiosis, during which diploid progenitor cells in mammalian gonads produce heritable haploid gametes, which are controlled by a series of strictly controlled gene expression events that determine key and highly coordinated cellular fates, including ovogenesis and spermatogenesis (Ying et al., 2000; Czerwoniec et al., 2009; Carreau and Hess, 2010; Lesch and Page, 2012; Miyamoto et al., 2012; Sahin et al., 2014).

The transforming growth factor beta-1 proprotein-like (TGF) -β family member proteins (e.g., BMP signal) are essential for primordial germ cells (PGC). They act as precursors of male and female gametes to produce oocytes and sperms. Directional interruption of Bmp2, Bmp4, Bmp8b, or BMP signal transducers, SMAD family member s1, 4 and 5, or protein kinase (ALK2) indicates a loss or decrease of PGCs (Lawson and Hage, 1994; Tremblay et al., 2001; McLaren, 2003; Saitou and Yamaji, 2012). In mammals, the PGC precursor is produced by equipotent epithelial cells, approximately on embryonic day 6 (E6), in response to Bmp4, Bmp8b, and Bmp2 signals emitted by the embryonic ectoderm and intraluminal endoderm (Chiquoine, 1954; Lawson et al., 1999; Loebel et al., 2003; de Sousa Lopes et al., 2004; Ohinata et al., 2009; van Werven and Amon, 2011). Fragilis, a member of interferon-(IFN) inducible transmembrane protein family, is a marker for early appearance of PGC in mice (Ying and Zhao, 2001). Starting from E7, designated PGCs in mice express various markers, including TNAP, SSEA1 and DPPA3 (Ginsburg et al., 1990; Sato et al., 2002; Avilion et al., 2003; Lence et al., 2016). Maintenance of the expression of multiple pluripotency genes, such as SRY- Sox2, Nanog and Oct4, is regulated by mouse PGCs (Yamaguchi et al., 2005; Chambers et al., 2007). Human PGC is first formed around the third week of pregnancy. It originates from precursor cells of the mesoderm, and depends on WNT and BMP pathways (Irie et al., 2015; Tang et al., 2016; Kojima et al., 2017). After formation, PGCs migrate and multiply through the back waves, and then enter the future genital ridge at approximately E7.5 to E10.5 (Anderson et al., 2000; Molyneaux et al., 2001; Richardson and Lehmann, 2010). The two germ cell-somatic signaling pathways of cKIT-STEEL and SDF-CXCR4 promote PGC multiplication and targeted migration (Larose et al., 2019). From the original band to future hindgut and genitals, cKIT-STEEL interactions are essential for PGC proliferation, survival and migration (Ohta et al., 2003; Ewen et al., 2009). In addition, SDF-1, which is expressed on the surrounding stromal genitalia, promotes PGC migration in a specific direction and can be detected by the PGC surface receptor, CXCR4 (Runyan et al., 2006; Gu et al., 2009). During the active migration process, venture capital continues to surge. At E10.5, there are about 500 PGCs in the genital ridge of each embryo. Sex determination begins during PGC migration to the genital ridge, and tends to differentiate into ovaries. Differentiation into testis is due to the effects of the Y-linked gene, SRY, on the XY genital ridge. The absence of such a gene for the XX genital ridge leads to the development of the ovary (Pepling and Spradling, 1998; Bowles and Koopman, 2010). Once prostaglandin cells enter the genital ridge, they are referred to as oogonocytes in females, or gonad cells in males. After colonization of the genital ridge, from E10.5 to E14.5, PGCs enter the mitotic proliferative phase. These embryonic germ cells undergo about 5 phases of mitosis and form “germline cysts” or “germ cell nests” due to incomplete cytokinesis (Pepling, 2006; Lei and Spradling, 2013). These “cysts” or “nests” eventually decompose and produce primary oocytes and spermatogonia in the, respectively, differentiated gonads (Larose et al., 2019).

### Spermatogenesis

**FIGURE 2:**
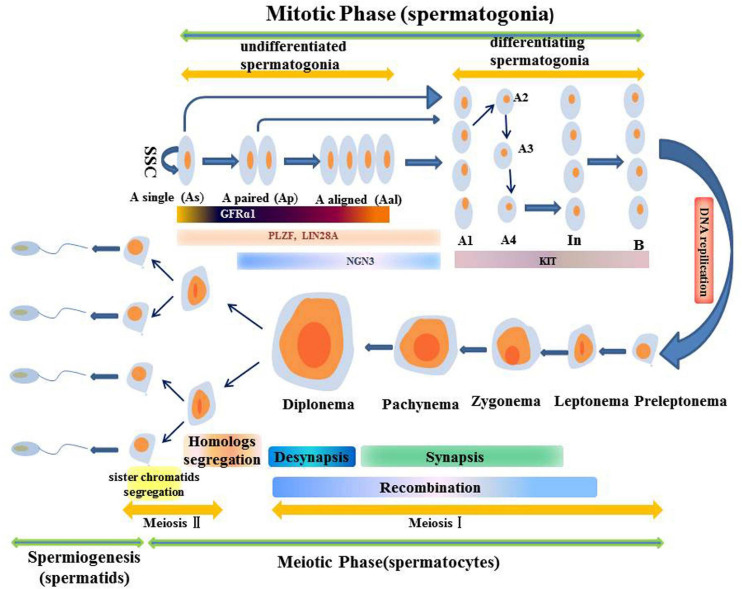
Schematic illustration of the spermatogenesis.

Sperm production is a complex asynchronous differentiation process that is divided into three stages based on the cell types found: Spermatogonia are formed through mitosis, spermatocytes are formed through meiosis, and the haploid stage of sperm cells or sperm formation (Shinohara et al., 2000; Wolgemuth et al., 2013; Griswold, 2016). Continuous sperm production depends on normal SSC functions. Spermatogonia with actual SSC functions can either self-renew to maintain the stem cell pool or can differentiate into Ap spermatogonia for spermatogenesis. The Ap spermatogonia stay connected through the intercellular bridge caused by incomplete cytokinesis. After repeated mitosis, AP spermatogonia divide to produce Aal spermatogonia (Lin and Tong, 2019). The Ap and Aal sperms are indiscriminate sperms, and many genes associated with self-renewal and proliferation, including Gfrα1, Nanos2, and Plzf among others are expressed in them to maintain the balance between self-renewal and differentiation. In addition, the GDNF/GFRα1/RET pathway is a key signaling pathway that regulates self-renewal and differentiation of undifferentiated spermatogonia. It can activate multiple signaling pathways such as the PI3K/AKT signaling pathway (Lin and Tong, 2019). Aal spermatogonia differentiates into A1 spermatogonia without mitosis. Then, they undergo a series of mitosis to produce A2, A3, A4, intermediate and B spermatogonia (Ohbo et al., 2003; Barroca et al., 2009; Nakagawa et al., 2010; Oatley and Brinster, 2012). Type A 1 to B Spermatogonia are referred to as “differentiated spermatogonia” (Battle, 2013). During the subsequent spermatogonia differentiation, genes such as Dnmt3b, Stra8, Kit and Ccnd2 among others are expressed. Among them, the genes associated with self-renewal are downregulated in undifferentiated sperms while those associated with differentiation are upregulated (Lin and Tong, 2019). The RA signal is essential for the differentiation of spermatogonia because it controls the expression of direct target genes (Hist1 cluster, Stra8 and Kit) (Lin and Tong, 2019). Type B spermatogonia pass through a long-lasting S phase and differentiate into pre-leptotene stage spermatocytes, followed by pre-meiotic stage I spermatocytes. This stage is highly regulated, and can be subdivided into four phases, that is, leptotene, zygotene, pachytene, and diplotene. The most complex and critical events of spermatogenesis, such as recombination and synapse, occur in the first stage before meiosis (Huckins, 1971; Handel and Schimenti, 2010). After prostatitis I, sperm cells are separated by two chromosomes, leading to doubling of round sperm cells. Spermiogenesis is the last stage of spermatogenesis, during which sperm cell nucleus undergo unique chromatin remodeling, including extreme compaction of the genome. Along with drastic reduction in nuclear volumes, its shape changes from round to rod-shaped, and finally pear-shaped, eventually forming mature elongated sperms (Ahmed and de Rooij, 2009) (Figure 2).

### Oogenesis

**FIGURE 3:**
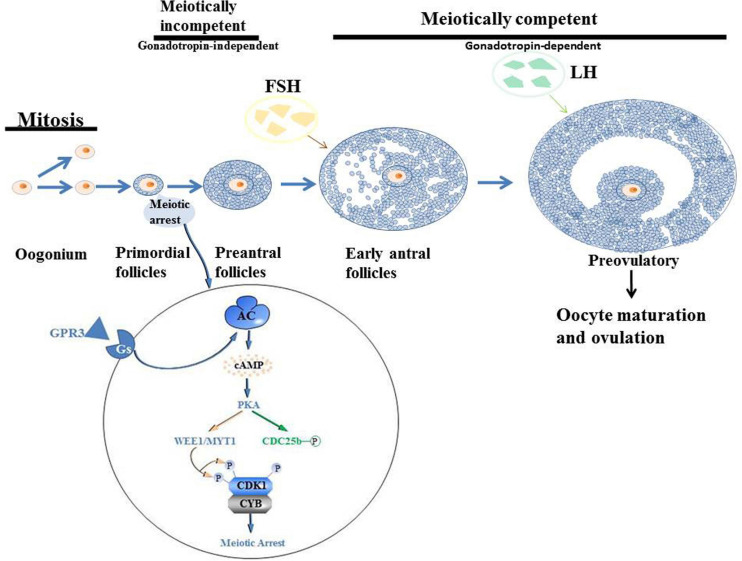
Schematic illustration of the oogenesis.

Oogenesis is a long-term process in mammals that begins in the embryonic period and ends in menopause. In oogenesis, two basic processes are necessary; the formation and maturation of oocytes and folliculogenesis. Follicles are reproductive units composed of oocytes, somatic cells and follicles. Oteocytes are made up of cumulus granulosa, mural granulosa and theca cells. In the embryonic stage, oogonia are the precursors of female gametes, expanding in number through mitosis. They differentiate into primary oocytes, grow in the follicle and undergo meiosis. Meiosis has a complex early stage that can be divided into five phases: leptotene, zygotene, pachytene, diplotene and diakinesis. Various key events, including homologous chromosome pairing, synapsis and recombination or crossover initially occur in the first period of the prophase (Andronico et al., 2019). In female mammals, meiosis has been shown to occur for a very long time. After the initiation of meiosis, oocytes are surrounded by a layer of granular cells that form the original follicles. When oocytes grow to more than 20 μm in size, granulosa cells become columnar and undergo mitosis to form multi-layered stratum granulosum (Sánchez and Smitz, 2012; Paci et al., 2018). Primordial follicles limit oocyte growth as well as the development of granulosa cells to seven layers. When oocytes progress from the diplotene phase, they enter a prolonged resting phase, referred to as the dictyate phase (Porras-Gómez and Moreno-Mendoza, 2017). Elevated cAMP levels in the oocyte determine meiosis arrest (Mehlmann, 2005). Oocytes activate Gs proteins through the G protein-coupled receptor 3 (GPR3) to stimulate cAMP production by AC (Mehlmann, 2005). cAMP-activated protein kinase A, promotes the phosphorylation (P) of cell cycle regulatory complex CDK1/cyclin B (CYB), leading to its inactivation. Through direct or indirect mechanisms, PKA leads to the phosphorylation and inactivation of CDC25b phosphatase (CDC25b-P) (Mehlmann, 2005). In addition, there is a possibility that PKA affects the activity of WEE1/MYT1 kinase. Phosphorylation of CDK1 by WEE1/MYT1 kinase leads to its inactivation, resulting in the failure to resume meiosis (Mehlmann, 2005). Elevated levels of LH hormone, produced by the pituitary, stimulate immature oocytes to resume meiosis (Liang et al., 1997; Rajkovic et al., 2004; Mehlmann, 2005; Porras-Gómez and Moreno-Mendoza, 2017). When oocytes complete the first meiosis phase and undergo cytoplasmic changes, they proceed to the metaphase II stage, at which time the oocyte mature (Figure 3).

**FIGURE 4:**
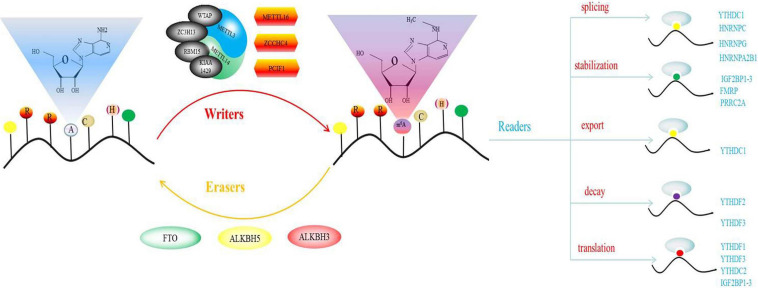
Schematic illustration of m^6^A.

**FIGURE 5:**
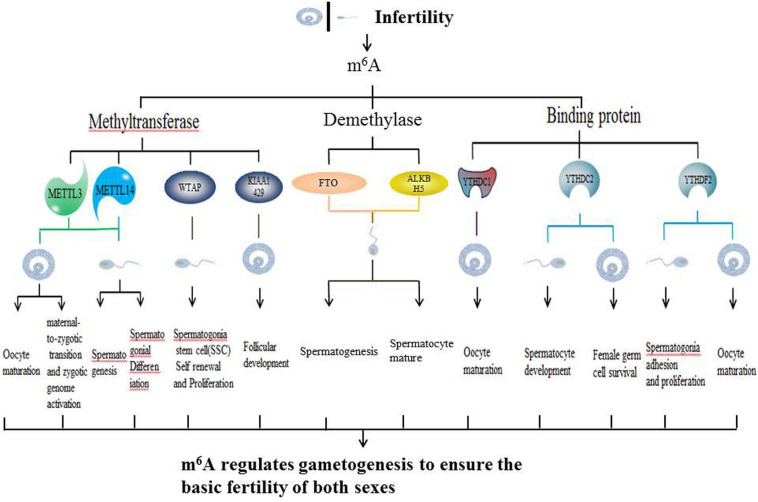
Schematic illustration of summarizing infertility and m^6^A.

In this review, we summarized up to date knowledge on studies of infertility in mammals. Normal gametogenesis is a basic guarantee of fertility, therefore, we discussed the impact of gametogenesis disorders on infertility. Then, we elucidated on RNA methylation m^6^A modification and its functions as a whole, briefly introduced m^6^A methyl transferase (“author”), dimethyl enzyme (“eraser”) and binding protein (“reader”) (Figure 4). Then, we discussed the roles of methyltransferase, demethylase and binding proteins in regulating gametogenesis (Figure 5 and Table 1). Finally, we evaluated the mechanisms through which m^6^A is involved in regulation of post-transcriptional gene expression as well as its role in infertility.

**TABLE 1:** m^6^A writer, eraser, binding protein, and gametogenesis.

M^6^A modulator	Source	Strain	Knockout treatment	Time of observation	Outcome	References
METTL3/METTL14	Mouse	C57BL/6J	Germ cell-specific inactivation of Mettl3 or Mettl14 with Vasa-Cre Com- bined deletion of Mettl3 and Mettl14 in advanced germ cells with Stra8-GFPCre	Postnatal days 5–7	Mice with single deletion of either Mettl3 or Mettl14 show normal spermatogenesis while Combined deletion of Mettl3 and Mettl14 disrupts spermatogenesis	Lin et al., 2017
METTL3	Mouse	CD1	Knocking down METTL3 with a microinjection of its specific siRNAs or morpholino into GV oocytes	3 weeks	Inhibition of oocyte maturation and defects in the maternal-to-zygotic transition	Sui et al., 2020
METTL3	Mouse	B6D2F1	CRISPR-Cas9 system	Postnatal days 6, 8, 10, and 12	Inhibit the differentiation of spermatogonia and block the initiation of meiosis	Xu et al., 2017
METTL3	Zebrafish	AB	TALEN mRNAs (300–500 pg) were microinjected into one-cell stage wild-type (WT) zebrafish embryos.	1–2 weeks	Gamete maturation fails and fertility decreases	Xia et al., 2018
WTAP	Mouse	C57BL/6N	Conditionally deleted the Wtap by crossing Wtap-floxed (Wtapfl/fl) and Amh-Cre mice	Postnatal days 14–180	Sterility and the progressive loss of the SSC population.	Jia et al., 2020
KIAA1429	Mouse	C57BL/6N	Kiaa1429fl/flmice were crossed with the Zp3-Cre mice	3 weeks	Female infertility with defective follicular development	Hu et al., 2020
ALKBH5	Mouse	C57BL/6J	Global inactivation of Alkbh5	Postnatal days 14 and 21	Male infertility	Tang et al., 2018
FTO	Mouse	GC-1 cells	CRISPR-Cas9 system	24 h	Chromosome-instability and G2/M Arrest	Huang T. et al., 2018
YTHDC1	Mouse	C57BL/6N	Ddx4-Creto inactivate Ythdc1 specifically	Embryonic days 8.5, 9.5, 11.5, and 15 3–6 weeks	Blocked primary follicular development	Kasowitz et al., 2018
YTHDC2	Mouse	C57BL/6	European Conditional Mouse Mutagenesis Program (EUCOMM)	Postnatal days 8, 10, 12, 14, and 30	Spermatocyte apoptosis	Abby et al., 2016
YTHDF2	Mouse	C57BL/6	GFP-precission-His 6-Flag-HA-HA epitope tag was inserted after the endogenous starting initiation ATG codon in exon 1 of Ythdf2.	2.5 days after priming and mating	Male-specific infertility	Ivanova et al., 2017
YTHDF2	Mouse	GC-1 cells	CRISPR/Cas9	8 h	Decreased cell proliferation and adhesion	Huang et al., 2020

## RNA Methylation m^6^A Modification

Both DNA and histone proteins can control gene expression through dynamic reversible chemical modification. Similar to DNA and proteins, RNA, which form the center of the central dogma transmit genetic information. Chemical modification of RNA molecules is more diverse and abundant when compared to DNA. Apart from the four basic bases, there are more than 170 chemically modified nucleotides, which play various roles in many different types of cellular RNAs, including rRNA, tRNA and mRNA, and snRNAamong others (Cantara et al., 2011; Boccaletto et al., 2018; Xuan et al., 2018). Approximately two-thirds of the discovered chemical RNA modifications involve the addition of methyl groups, and these modifications are typically introduced in a post-transcriptional modification reaction. Co-transcriptional modifications are very few (Cantara et al., 2011).

Among these modifications, N6-methyladenosine (m^6^A) exerts the most significant effect on gene expression, and is the first modification that has been shown to regulate mRNA abundance. N6-methyladenosine (M^6^A) modification was first described 40 years ago. M^6^A was first discovered in mouse L cell polyA-RNA, and was subsequently confirmed to be the most widely distributed and highest internal modification in mRNA (Schäfer, 1982). It was first detected in mRNA isolated from eukaryotes and in viral RNA replicating in the nucleus (Zhao and He, 2015). Methylation has also been shown to occur in the intron region of pre-mRNA, confirming that methylation occurs shortly after co-transcription or transcription (Roundtree et al., 2017a). m^6^A modification can modulate mRNA fate at splicing, stability, subcellular localization, output, decay, and transformation levels (Dominissini et al., 2012; Wang et al., 2015). m^6^A, which is ubiquitous in many transcripts, is uniquely and conservatively distributed. The consensus sequence, RRACH, has the highest frequency of occurrence in m^6^A, where R represents purine, A represents m^6^A site, while H represents a non-guanine base. However, the M^6^A on RRACH is not randomly distributed on the entire report card. It mainly appears in the coding sequence (CDS) of 3′-UTR, especially in the area near the stop codon (Perry et al., 1975; Wan et al., 2015). About 0.1–0.4% of adenosine residues in cellular mRNA are m^6^A. The average content of m^6^A is estimated to account for 3–5 residues in mammalian mRNA, 1–15 residues in RSV RNA, and 1.4–2.0 residues in Arabidopsis (Heck and Wilusz, 2019). M^6^A regulates RNA gene expression and metabolism through the action of methyltransferase (writer), demethylase (eraser) and m^6^A binding protein (reader). Demethylase is a proof that m^6^A modification is dynamically reversible.

The characteristics of these effectors in various biological systems emphasize the versatility and tunability of their functions, and confirms that the local environment is an important determinant of their biological effects (Yue et al., 2015). For example, reproductive functions of mammals have a very high probability of being affected by acquired dispositions, such as from the external environment, drugs and heavy metals. Therefore, these factors have an impact on epigenetic m^6^A modification, which in turn affect reproductive functions, leading to infertility (Figure 4).

### M^6^A Methyltransferase

Methyltransferase complexes, which consists of two sub-complexes (MAC and MACOM) catalyzes m^6^A modification. MAC is the core component of MTC. It is composed of METTL3 and METTL14 and is a heterodimer with a size of 200 kDa (Liu et al., 2014; Wang et al., 2016). Both METTL3 and METTL14 have methyltransferase structures. METTL14 is degenerate compared to METTL3 and has important implications for the stability of METTL3 conformation and binding of the RNA substrate. METTL3 is the only subunit that requires the donor substrate, *S*-adenosylmethionine, to bind the catalytic site to exert its catalytic activity, which involves mRNA biogenesis, decay and translation (Śledź and Jinek, 2016; Choe et al., 2018). METTL3 and METTL14 are essential in properly maintaining m^6^A in the body. Abnormal expressions or mutations of METTL 3 and/or METTL 14 are associated with the development of various human diseases, including infertility. MACOM contains WTAP and its cofactor, KIAA1429. KIAA1429 plays an important role in maintaining the metabolic capacity of oocytes (Ping et al., 2014; Schwartz et al., 2014; Hu et al., 2020). HAKAI, RBM15 and ZC3H13 anchor the MTC to nuclear speckles and U-rich regions near the m^6^A site (Patil et al., 2016; Knuckles et al., 2018; Wen et al., 2018; Akichika et al., 2019). A new methyltransferase, PCIF1, has been shown to affect the methylation of adenosine N^6^ sites. However, PCIF1 is not involved in internal m^6^A methylation, it mainly catalyzes m^6^AM methylation of mRNA (Pendleton et al., 2017; Sun et al., 2019). Two other methyltransferase proteins, METTL16 and ZCCHC4 have also been reported. METTL16 can catalyze U6 splicing of RNA and m^6^A in some structural RNAs, while ZCCHC4 can mediate rRNA methylation in the AAC motif (Brown et al., 2016; Warda et al., 2017; Mendel et al., 2018; Ma et al., 2019).

### M^6^A Demethylases

Fat-mass and obesity-associated protein was the first m^6^A demethylase to be discovered in 2011. As a result, the mechanisms and biological functions of m^6^A have been widely evaluated. The second m^6^A demethylase, ALKBH5, was discovered in 2013 (Zheng et al., 2013). These demethylases can exert a series of complex intermediate reactions to reverse the m^6^A methylation process of mRNA. The priority target of FTO is m^6^Am instead of m^6^A (Jia et al., 2011). Unlike FTO, knocking out ALKBH5 elevates m^6^A levels but not m^6^Am, implying that ALKBH5 selectively demethylates m6A, but has no affinity for m^6^Am. In addition, ALKBH5-deficient male mice were shown to exhibit abnormal fertilization characteristics. This was associated with abnormal sperm production and apoptosis in mouse testicles. Another m^6^A demethylase, ALKBH3, has been shown to be common in tRNA than in mRNA and in rRNA (Ueda et al., 2017). We believe that there are more m^6^A demethylases that are yet to be discovered.

### M^6^A Binding Proteins

Discovery of the m^6^A binding protein provides a basis for studying the functions of m^6^A modification. Currently, two different types of m^6^A binding proteins have been characterized. This characterization is based on the mechanism of recognizing and binding RNA with m^6^A markers. From a structural aspect, binding proteins include YTH YTHDF1-3 and YTHDC1-2 (Meyer et al., 2015). In addition to the yth domain protein, eIF3 is also a binding protein for m^6^A, which promotes hat-independent translation when inducing cellular stress (Alarcón et al., 2015). There are two types of heteronuclear ribonucleoproteins; HNRPA2B1 and HNRNPC. In addition, IGF2BP1-3 was has also been reported (Liu et al., 2015; Huang H. et al., 2018). Other m^6^A binding proteins, such as ABCF1, FMRP, ELAV1, PRRC2A, and G3BPs have also been discovered (Wu et al., 2019). It has been shown that in pre-mRNA, m^6^A, YTHDC1, HNRNPC, HNRNPG, and HNRPA2B1 are involved in the regulation of mRNA splicing (Mauer et al., 2017; Zhou et al., 2019). Moreover, YTHDC1 can mediate the export of processed RNA from the nucleus to the cytoplasm (Roundtree et al., 2017b). YTHDC2 or YTHDF2, and YTHDF3 bind transcripts containing m^6^A, thereby causing it to be degraded in P bodies in the cytoplasm (Li et al., 2017). PRRC2A, FMRP and IGF2BP1/2/3 play the opposite role, and have been shown to be important in maintaining the stability of m^6^A modified transcripts (Wang et al., 2014). The combination of YTHDC2, YTHDF1 and m^6^A promotes mRNA translation. In addition, YTHDF3 and IGF2BP1/2/3 play the same role (Xiao et al., 2016; Hsu et al., 2017; Li et al., 2017; Shi et al., 2017; Zhao and He, 2017; Zhang et al., 2018).

## M^6^A Modification and Gametogenesis Disorders

**FIGURE 6:**
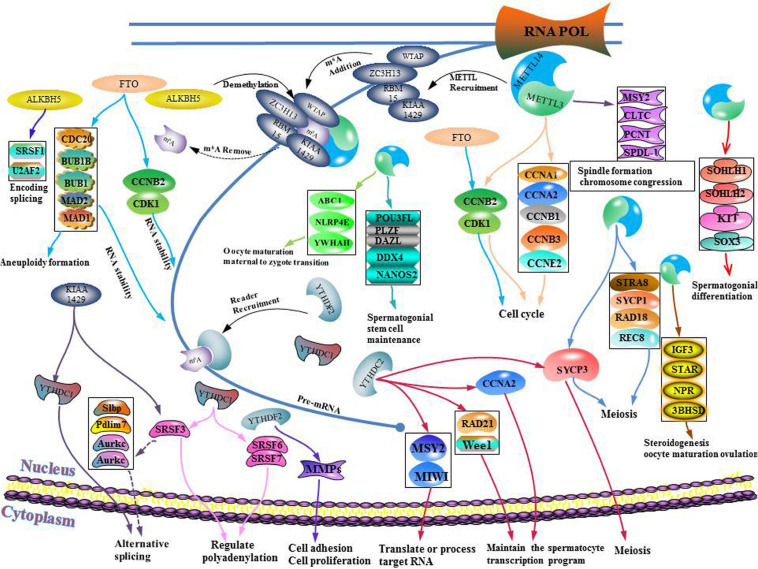
Schematic illustration of the molecular mechanism between m^6^A writer, eraser, binding protein, and gametogenesis disorder.

Sexual reproduction begins when parent gametes form functional gametes through meiosis, and ends with the formation of oosperms. In this extremely complex and highly precisely regulated process. Sperm production requires a high degree of coordination between transcription and conversion levels. Therefore, there are extremely complex regulatory procedures in this process, which ensure that spermatogenic cells at different developmental stages can correctly express specific gene sets (Lin et al., 2017). Spermatogenesis exhibits various features that are divided into different phases. First, the transcription program is turned on. The time at which the transcriptional program is started may be earlier when compared to the time at which the biological process is finally determined. The second phase involves the detection a higher level of alternative spliceosomes in spermatogenic cells. In the third phase, the transcriptional activity is significantly reduced in the early stages of meiosis I and stopped in the latter stages of spermiogenesis. Oocyte maturation is also a key step in sexual reproduction. It is directly related to the quality of oocytes and the subsequent reproductive processes (Mehlmann, 2005). Since transcriptional activities of oocyte DNA are inhibited during cell maturation, and the whole genome of the oocyte is only reactivated at the metaphase blastocyst stage, it is important to precisely regulate post-transcriptional levels of intracellular mRNA during oocyte maturation (Mehlmann, 2005). m^6^A plays an important role in different gametogenesis stages (Table 2 and Figure 6), however, it has not been established whether they are closely related. For example, a decrease in METTL3 affects the addition of m^6^A. In cases of decreased m^6^A, downstream YTHDC2 may not perform its normal mRNA translation functions, thereby affecting germ cell proliferation and differentiation. More studies should be performed to elucidate on these relations. Manipulation of specific m^6^A regulators maybe potential therapeutic targets. A limited number of studies have evaluated the role of m^6^A regulator inhibitors in infertility. These inhibitors may provide a scientific basis for targeted treatment of infertility.

**TABLE 2:** Role of m^6^A modulator on infertility.

m^6^A modification	Modulator	Physiological process	Molecular mechanism	References
m^6^A methyltransferase	METTL14/METTL3	Spermatogenesis	METTL3 and Mettl14 combined regulate the coordinated translation of different stages of spermatogenesis	Lin et al., 2017
	METTL3	Spermatogonial differentiation	METTL3 promotes the differentiation of spermatogonia and regulates the initiation of meiosis and alternative splicing of mRNA	Xu et al., 2017
		Oocyte maturation maternal-to-zygotic transition and zygotic genome activation	METTL3 enables high expression of mRNA in oocytes and promotes the secretion of 11-ketotestosterone and 17β-estradiol Knocking down Mettl3 perturbed meiotic progression and disrupted spindle formation and chromosome movement, consequently inducing the high frequency of aneuploidy in oocytes.	Xia et al., 2018; Sui et al., 2020
	WTAP	Spermatogonia stem cell (SSC) Self renewal and Proliferation	Alternative splicing events of transcripts encoding SSC niche factors were sharply altered and translation of these transcripts were severely dysregulated by Wtap deletion.	Jia et al., 2020
	KIAA1429	Follicular development	KIAA1429 regulates mRNA levels and alternative splicing of mRNA in oocytes	Hu et al., 2020
m^6^A demethylases	ALKBH5	Spermatocyte mature	ALKBH5 ensures the correct splicing of long 3′-UTR transcripts, regulates meiosis-related genes such as Sycp1, Sycp2 and Marf1, and promotes meiosis.	Tang et al., 2018
	FTO	Spermatogenesis	FTO directly regulates the expression of the core MCC components and G2/M regulators through the m^6^A/RNA decay pathway, thus regulating cell cycle and mitosis checkpoint in spermatogonia.	Huang T. et al., 2018
	YTHDC1	Oocyte maturation	YTHDC 1 regulates the splicing of mRNA in oocytes to make the 3′ untranslated region of the appropriate length and promote the growth and maturation of oocytes	Kasowitz et al., 2018
m^6^A binding proteins	YTHDC2	Spermatocyte development	YTHDC2 stabilizes the transcription during prophase I of meiosis and correctly induces the meiosis program	Abby et al., 2016
		Female germ cell survival	YTHDC2 regulates the normal progression of oocyte meiosis through post-transcriptional	Bailey et al., 2017
	YTHDF2	Spermatogonia adhesion and proliferation	YTHDF2 affects the adhesion and proliferation of spermatogonia by regulating the expression level of matrix metallopeptidase (MMPs)	Huang et al., 2020
		Oocyte maturation	YTHDF2 regulates the appropriate transcript dosage during oocyte maturation and promotes the degradation of oocyte mRNA	Ivanova et al., 2017

### M^6^A Methyltransferase and Gametogenesis Disorders

The methyltransferases (METTL3 and METTL14) are modulators that regulate germ cells. After knocking out METTL3 or METTL14, it was found that m^6^A levels in undifferentiated spermatogonia were significantly suppressed (Lin et al., 2017). Moreover, undifferentiated spermatogonia in METTL3 or METL14 knockout testis were found to exhibit obvious translational disorders. Before the appearance of obvious morphological defects, mRNA levels of undifferentiated spermatogonia were found to have been altered. These findings indicate that m^6^A modification mediated by METTL3/METTL14 affects the fate of SSC (Lin et al., 2017). The knockout of either METTL3 or METTL14 genes by Stra8-GFPCre in mice did not exert any effect on spermatogenesis. Combined knockout of METTL3 and METTL14 in mice was shown to lead to normal meiosis, but abnormal spermatogenesis (Lin et al., 2017). These results imply that m^6^A requires the involvement of METTL3 and METTL14 to timely regulate the translation of methylation transcription and the production of proteins required for spermatogenesis (Lin et al., 2017).

As part of the methyl transferase complex, METTL3 was the first to be discovered. METTL3 was found to modulate neurological functions in mice and sex determination in Drosophila (Chen et al., 2018), and had an important impact on the pluripotency of stem cells and early embryonic development in mice (Aguilo et al., 2015; Geula et al., 2015; Haussmann et al., 2016). Moreover, it has an important effect on gamete maturation and fertility of female zebrafish (Xia et al., 2018). In their study, Sui et al. microinjected siRNA targeting METTL3 into GV oocytes to knock down METTL3. They found that meiotic maturation of mammalian oocytes is modulated by MEtTL3-mediated m^6^A (Sui et al., 2020). Spindle formation and chromosomal aggregation occur during the maturation of mouse oocytes. These processes require the expression of genes such as cltc, Msy2, Pcnt and Spdl-1. Western blot analysis revealed that their protein abundance decreased after METTL3 knockdown (Sui et al., 2020). After METTL3 knockdown, HPG incorporation experiments revealed that the translation efficiency of maternal mRNA in oocytes was suppressed, implying that oocyte maturation can be affected by mRNA translational efficiency. When the efficiency is low, oocyte maturation is inhibited, which leads to defects in mother-to-enzyme transition (Sui et al., 2020).

In addition, METTL3 has been shown to regulate the maturation process of oocytes by regulating the mRNA levels of oocytes. In METTL3 cKO zebrafish, oocytes were found to stagnate during early development while the follicular maturation rate was significantly low than that of normal zebrafish (Xia et al., 2018). Suppressed m^6^A modification levels in Zmettl3 m/m zebrafish oocytes lacking METTL3 was found to lead to abnormal expressions of key genes associated with sex hormone synthesis and gonadotropin signal transduction in zebrafish. In turn, levels of 11-ketotestosterone and 17-β-estradiol, secreted by offspring embryos, were found to have significantly decreased, which led to impaired gamete maturation disorders and weakened fertility (Xia et al., 2018).

Testicular spermatogonia of METTL3 cKO mice did not reach the pachytene phase of meiosis (Xu et al., 2017). Xu et al. proved that m^6^A mediated by METTL3 affects spermiogenesis, which is crucial to male fertility. They found that spermatogonia differentiation and spermatocyte meiosis processes during spermatogenesis are mediated by METTL3 (Xu et al., 2017). Transcriptome analysis and qRT-PCR analysis revealed that after METTL3 knockout, there was an alternative splicing dysregulation that was not conducive for spermatogenesis (Xu et al., 2017). Four types of genes related to spermatogenesis were identified the METTL3 cKO testis. These genes regulate cell cycles, sperm stem cell maintenance, spermatogonia differentiation and spermatocyte meiosis, respectively (Xu et al., 2017). They were significantly down-regulated after METTL3 knockout. Genes that regulate the cell cycle include Ccna1, Cdk1, and RAD51 among others; genes involved in maintenance of spermatogonial stem cells include Dazl, Plzf and Bcl6 among others; genes associated with differentiation of spermatogonia include Sohlh1, Kit and Sox3 among others; genes involved in meiosis include Stra8, Sycp3, and Smc1b. After METTL3 knockout, initial spermatogonia differentiation and spermatocyte meiosis were found to be severely blocked. Therefore, METTL3 significantly affects sperm production from undifferentiated sperms. This outcome is attributed to significant suppression of the abundance of STRA8-positive cells and synaptic complex protein SYCP3 levels (Xu et al., 2017).

A highly effective and selective METTL3 inhibitor, STM2457, has been reported. This small molecule inhibitor is highly specific for METTL3, but has no inhibitory effects on other RNA methyltransferases (Yankova et al., 2021). Various chemical and biological molecules that can affect METTL3 have also been identified. For example, cigarette smoke condensates induce METTL3 overexpression. The intestinal microbial metabolite, butyrate, promotes the downregulation of METTL3. Several micro-RNAs, including miR-186, miR-4429, and miR-600 have also been shown to target METL3 mRNA, inhibiting its expression (Zeng et al., 2020).

Wilms tumor 1-associated protein is a subunit of m^6^A-METTL related complexes. During spermatogenesis, its gene was found to be significantly expressed in mouse Sertoli cells. In Sertoli cells, WTAP was found to significantly affect SSC proliferation and self-renewal (Jia et al., 2020). By conditionally eliminating WTAP in supporting cells, it was found that SSC was progressively lost and eventually led to infertility (Jia et al., 2020). WTAP knockout was also found to lead to progressive loss of SSC and depletion of germ cells, which reduced sperm concentrations in mice, thereby contributing to the sterile phenotype of mice. Moreover, by making WTAP ineffective, expression levels of GDNF, which is essential for maintaining the SCC pool, were also found to be down-regulated, therefore, sertoli cells cannot maintain the SSC pool, leading to SSC depletion. In addition, transcripts associated with SCC maintenance and spermatogonia differentiation in sertoli cells are severely affected by gene transcription and translation changes (Jia et al., 2020). Knockout of WTAP was found to alter splicing events in sertoli cells, with a large number of abnormal splicing events occurring in m^6^A-rich genes. If the WTAP function is lost, splicing events in genes that regulate spermatogenesis are altered, which eventually affects spermatogenesis. The WTAP-mediated m^6^A regulates mRNA transcription and translation, thereby coordinating the expression of essential genes, so that SSC maintenance of sertoli cells and differentiation of spermatogonia occur normally (Jia et al., 2020).

The downregulation and overexpression of METTL3 protein leads to upregulation of WTAP. In particular, METTL3 levels regulate the expression of WTAP at multiple levels through direct and indirect mechanisms, including mRNA translation and stability (Sorci et al., 2018). Therefore, the dynamic balance of WTAP expression is indirectly maintained through METTL3 to ensure normal progression of SSC proliferation and self-renewal, which is essential for preventive treatment of spermatogenesis disorders.

Virus-like m^6^A methyltransferase associated protein is also an important component of m^6^A-METTL related complex. It has been shown to mediate the deposition of m^6^A in the 3′UTR and the region close to the stop codon. It also affects the alternating polyadenylation in HeLa cells. KIAA1429 has been found to be highly expressed in mouse oocytes, implying that it plays a specific function in mouse oocytes (Hu et al., 2020). Hu et al. (2020) found that the expression of KIAA1429 mRNA in GV oocytes is dominant, and that the role of KIAA1429 during oocyte growth is essential for female fertility. HE staining and TUNEL analysis confirmed that the expression of KIAA1429 is essential for folliculogenesis. The loss of KIAA1429 function in oocytes is associated with follicular growth defects, leading to abnormal proliferation and apoptosis of granulosa cells (Hu et al., 2020). KIAA1429cKO mice were found to exhibit multiple oocyte follicles. In addition, compared to the control group mice, there were no significant differences in the number of follicles at different stages. KIAA1429cKO female mice have abnormal follicular morphologies. In preovulatory follicles, abnormal RNA granules were found in the cytoplasm of KIAA1429cKO oocytes, and the GV oocytes were much smaller than those of the control group (Hu et al., 2020). Moreover, KIAA1429 is essential for oocyte competence in mediating chromatin configuration and for GV oocyte RNA metabolism (Hu et al., 2020). Srsf3 mediates Pdlim7 exon inclusion bodies to maintain proper GVBD during meiosis in mouse oocytes (Hu et al., 2020). KIAA1429 may also affect the location of YTHDC1 through Srsf3 to regulate alternative mRNA splicing. Therefore, it is involved in post-transcriptional regulation, promotion of follicular development and in ensuring fertility in women.

### M^6^A Demethylases and Gametogenesis Disorder

Fat-mass and obesity-associated protein is a member of the α-ketoglutaric acid dependent dioxygenase alkb family. It is encoded by fat and obesity-related genes located on human chromosome 16 (Huang T. et al., 2018). The reduction in semen quality among azoospermia patients has been attributed to the occurrence of two missense mutations in FTO. Huang et al. found that by knocking out FTO, the expression levels of MCC in spermatogonia were upregulated, thereby inhibiting chromosomal segregation and inducing aneuploidy. Moreover, the expression of CDK1 and CCNB2 were found to be significantly downregulated in FTO-KO cells. FTO was shown to regulate G2/M transition under the influence of the Cdk1/Ccnb2 complex, and expression levels of CDK1 and CCNB2 were significantly downregulated in FTOcKO cells, implying that FTO knockout can cause chromosomal instability in mouse spermatogonia and arrest the G2/M transition (Huang T. et al., 2018).

Several inhibitors of FTO have been identified targets for Rhein was the first effective FTO inhibitor to be identified. However, its selectivity is not strong and it also exhibits a certain inhibitory activity against ALKBH5. Entacapone has also been identified as a potential FTO inhibitor. As a drug for the treatment of Parkinson’s disease, it may have a certain effect on metabolic disorders (Zhao et al., 2020). Studies on m^6^A target inhibitors in infertility are limited. Some FTO inhibitors may have potential therapeutic effects on infertility. For example, Meclofenamic acid (MA) is a non-steroidal anti-inflammatory drug that is considered to be a highly selective FTO inhibitor. As an ethyl ester MA derivative, MA-2 can effectively inhibit the growth of glioblastoma stem cells (GSC) and tumor formation induced by GSC. It also has a significant inhibitory effect on the expression of FTO (Zhao et al., 2020).

Alk B homolog 5 has been found to be the second M^6^A demethylase with the highest expression levels in mouse testis. Its deletion was found to significantly elevate m^6^A levels in testicular cells. Male mice lacking ALKBH5 exhibit abnormal spermatogenesis, leading to spermatogonia apoptosis and formation of abnormal sperms, which are not fertile. Meiosis and haploid phases of spermatogenesis were found to be significantly affected by ALKBH5 in cKO mouse (Mauer et al., 2017). The 3′-UTR mRNA length affects the enrichment and levels of m^6^A. A longer 3′-UTR mRNA has elevated m^6^A levels than the shorter 3′-UTR mRNA. In the longer 3′-UTR mRNA, M^6^A is enriched in the 3′-UTR region near the stop codon. ALKBH5 knockout leads to abnormal splicing of the genes that encode splicing factors such as Khdrbs3, Sfswap, Snrnp70, Srsf1 and U2af2. Due to abnormal splicing of these genes, more target gene splicing errors occur, then, a vicious cycle of abnormal splicing occurs, amplifying the initial adverse effects. In mature spermatocytes and round spermatocytes, deletion of ALKBH5 can lead to abnormal splicing of long 3′-UTR mRNA while the short mRNA is produced with increasing m^6^A levels, indicating that the correct splicing of long 3′-UTR mRNA may require ALKBH5-mediated m^6^A demethylation (Tang et al., 2018). The m^6^A of Alkbh5 cKO spermatocytes cannot be demethylated while various genes, such as ycp1, Sycp2 and Marf1, which are essential for meiosis are dysregulated, leading to meiotic defects.

### M^6^A Binding Proteins and Gametogenesis Disorder

YTH domain-containing 1 has a significant regulatory effect on the development of spermatogonia and oocyte growth and maturation. In oocytes, the failure of YTHDC1 blocks the primary follicular phase. Kasowitz et al. found that 3′-terminal pro-mRNA processing factors (CPSF6, Srsf3, and Srsf7) are closely associated with YTHDC1, a key nuclear factor in the processing of precursor mRNA transcripts. Knockout of YTHDC1 changes the length of the 3′UTR. This is because, extensive selective polyadenylation occurs in oocytes, leading to alternative splicing defects in oocytes.

GO analysis showed that YTHDC2 in mouse testes is highly expressed. *In vitro* pulldown assays proved that YTHDC2 preferentially binds m^6^A (Abby et al., 2016). After YTHDC2 knockout, both male and female mice exhibited infertility, implying that YTHDC2 is essential for fertility. In YTHDC2cKO mice, germ cells could not develop to the zygotene stage of meiotic prophase I (Abby et al., 2016). YTHDC2cKO mice have normal spermatogonia and sertoli cells, indicating that YTHDC2cKO mice only exhibit spermatogenesis defects at the spermatocyte stage (Abby et al., 2016). To stabilize transcription in the prophase I of meiosis, and to ensure that meiosis is correctly induced, YTHDC2 interacts with MEIOC in an RNA-independent manner. Therefore, YTHDC2 is necessary for normal development of spermatocytes (Abby et al., 2016). YTHDC2 interacts with RNA particles and post-transcriptionally regulates the meiotic process of germ cells by binding Ccna2, other mitotic transcripts and specific piRNA precursors (Bailey et al., 2017). Adult females with YTHDC2cKO were also found to have thin uterine walls and small ovaries, without developed follicles in the ovaries, implying that YTHDC2 knockout in female mice leads to infertility (Bailey et al., 2017). Moreover, YTHDC2cKO female mice were found to have much less DDX4 and germ cells in the ovaries at birth (P0.5), no primordial follicles were detected in the ovaries at postnatal 5 days (P5), and no germ-vesicles were detected on P21. These findings suggest that YTHDC2 is essential for female germ cell survival during the embryonic period (Bailey et al., 2017). After YTHDC2 knockout, germ cells were found to exhibit a nuclear morphology that is similar to that of premeiotic cells without chromosomal changes in the prophase stage of meiosis. Without YTHDC2 regulation, female germ cells cannot correctly reach the pre-meiotic phase in the fetal ovary (Bailey et al., 2017).

YTHDF2 is also involved in degrading target mRNA. YTHDF2 is differentially expressed in tissues, with the testis exhibiting the highest expression levels, especially during spermatogenesis. YTHDF2cKO male mice have been shown to be fertile with normal seminiferous tubule histologies. After YTHDF2 knockout, MMPs were found to be downregulated, affecting cell adhesion and proliferation. Edu analysis revealed that the positive rate of Edu in the Wild type (WT) group was significantly higher than that in the YTHDF2cKO group, implying that proliferation of spermatogonia is inhibited after YTHDF2 knockout. Morphologically, wild-type spermatogonia are polygons while YTHDF2cKO spermatogonia are fusiform or round. Cell-adhesion assays revealed that YTHDF2 has a significant effect on the adhesion and spread of spermatogonia (Huang et al., 2020).

YTHDF2 has the ability to regulate oocytes to reach metaphase stage of meiosis II. During early zygotic development, these oocytes can remain in the metaphase stage of meiosis II. Large amounts of YTHDF2 in GV and MII oocytes imply that it is also expressed during oocyte maturation, spermatogenesis or follicular development. YTHDF2 has been detected in the cytoplasms of germ cells and somatic cells (Ivanova et al., 2017). When the corpus luteum is found in the ovary of YTHDF2cKO female mice, it indicates that ovulation has taken place and that that woman is infertile. Therefore, YTHDF2 knockout leads to female-specific infertility (Ivanova et al., 2017). By producing YTHDF2 maternal conditional deletion (mCKO) oocytes, without affecting the expression of YTHDF2 in somatic granulosa cells, YTHDF2 has been proven to be indispensable for women to have children. MII oocyte analysis showed that after YTHDF2 knockout, oocyte growth or the formation of maternal transcriptome was not seriously affected. In the process of oocyte maturation, YTHDF2 is needed to inform the appropriate transcript dosage (Ivanova et al., 2017).

## Discussion

With developments in methylation modifications of RNA, studies on RNA methylation modification have gradually developed from relatively concentrated functional research to studies on disease. To maintain generations, the harm caused by reproductive health and its influencing factors to humans should be urgently resolved. RNA methylation modification is an important epigenetic phenomenon. M^6^A modification and its methyltransferase and demethylase, binding protein have an extremely important impact on gametogenesis.

We have discussed the role and mechanism of RNA methylation modification in infertility. The mechanism involves the cooperation of METTL3 and METTL14 to control timely translation of methylation transcription, thereby, appropriately regulating the production of proteins that are necessary for spermatogenesis. METTL3 regulates the differentiation of spermatogonia and plays an important regulatory role in oocyte maturation. KIAA1429 plays a vital role in follicle formation and has a regulatory on normal spermatogenesis. The correct regulatory mechanism of YTHDC2 on meiosis in gamete development has not been clearly elucidated. YTHDF2 regulates spermatogonia proliferation and has a role in the maturation of oocytes.

Secondly, by identifying specific m^6^A regulators, potential preventive and treatment methods for infertility can explored. Proteins for m^6^A regulation can be used as biomarkers to reflect gametogenesis disorders in both sexes and to achieve preventive treatment of infertility. Precise treatment of gene targets such as METL3, WTAP, KIAA1429, FTO, ALKBH5, YTHDC1, YTHDC2, and YTHDF2, by regulating m^6^A modification ensures normal gametogenesis and exerts a better therapeutic effect on infertility. Entacapone, MA drugs, STM2457, butyrate, miR-186, miR-4429, miR-600 and let-7G, Rhein, MA-2 inhibitors, as well as folate, betaine and methionine methylation donors can be used in the treatment plans for gametogenesis disorders.

RNA methylation can prevent and treat infertility by regulating gametogenesis. This discovery has important research significance in theory and practice. However, the mechanisms involved have not been clearly established. For example, it has not been determined whether the specific molecular mechanism that causes infertility is associated with other diseases. Moreover, it has not been determined whether other RNA methylation modification proteins affect gametogenesis. To comprehensively understand the regulatory mechanisms of RNA methylation on infertility, studies should determine how RNA methylation affects gametogenesis under the stimulation of the external environment, drugs and poisons.

## Author Contributions

XL, HW, BL, ZQ, JL, BX, WL, and ZX: revising the manuscript. YD: conception and design, revising it critically for important intellectual content, and final approval of the version to be published. All authors substantially contributed to the review and read and approved the final manuscript.

## Conflict of Interest

The authors declare that the research was conducted in the absence of any commercial or financial relationships that could be construed as a potential conflict of interest.

## Publisher’s Note

All claims expressed in this article are solely those of the authors and do not necessarily represent those of their affiliated organizations, or those of the publisher, the editors and the reviewers. Any product that may be evaluated in this article, or claim that may be made by its manufacturer, is not guaranteed or endorsed by the publisher.

## Abbreviations

WHO: World Health Organization
PGCs: Primordial germ cells
BMP: bone morphogenetic protein
TNAP: tissue non-specific alkaline phosphatase
SSEA1: stage-specific embryonic antigen 1
DPPA3 or STELLA: developmental pluripotency type 3 protein
SRY: sex determining region-Y
Sox2: sex determining region-Y -box2l
SDF-1: stromal cell-derived factor 1
CXCR4: C-X -C motif chemokine receptor 4
SSCs: spermatogonial stem cells
As: single spermatogonia
Ap: A-paired spermatogonia
Aal: A aligned spermatogonia
Gfr α 1: glial cell line-derived neurotrophic receptor α 1
Nanos2: anos homolog 2
Plzf: Zbtb16
Id4: inhibitor of differentiation
Ngn3: Neurog 3
RA: retinoic acid
Hist1 cluster: replication-de pendent core histone genes
FF: follicular flfluid
AC: adenylate cyclase
PKA: cAMP activates protein kinase A
CYB: cyclin B
LH: luteinizing hormone
rRNA: ribosomal RNA
tRNA: transfer RNA
mRNA: messenger RNA
snRNA: small nuclear RNA
m^6^A: N6-methyladenosine
polyA-RNA: polyadenylated RNA
RSV: Rous Sarcoma virus
CDS: coding sequences
3 ′ -UTRs: 3 ′ -untranslated regions
MTC: methyltransferase complexes
MACOM: m^6^A-METTL related complex
METTL3: methyltransferase-like protein 3
METTL14: methyltransferase-like protein 14
SAM: *S-*adenosylmethionine
WTAP: Wilms tumor 1-associated protein
KIAA1429: virus-like m^6^A methyltransferase associated protein
HAKAI: Cbl proto-oncogene E3 ubiquitin protein ligase-like1
RBM15: RNA binding motif protein 15
ZC3H13: zinc finger CCCH domain-containing protein 13
PCIF1: Phosphorylated CTD Interacting Factor 1
m^6^Am: N6-2-O-dimethyladenosine
METTL16: methyltransferase-like protein 16
ZCCHC4: CCCH domain-containing protein 4
FTO: Fat-mass and obesity-associated protein
ALKBH5: Alk B homolog 5
ALKBH3: Alk B homolog 3
YTHDF1-3: YTH N6-methyladenosine RNA binding protein 1-3
YTHDC1-2: YTH domain-containing 1-2
eIF3: eukaryotic initiation factor 3
HNRNP: heterogeneous nuclear ribonucleoprotein
HNRNPC: heterogeneous nuclear ribonucleoproteins C
IGF2BP1-3: insulin-like growth factor 2 mRNA-binding proteins 1-3
ABCF1: ATP binding cassette subfamily F member 1
FMRP: fragile X mental retardation protein
ELAV1: ELAV-like protein 1
PRRC2A: proline rich coiled-coil 2A
AS: alternative splicing
Zmettl3 m/m: zygotic deficiency mutant lines
APA: alternative polyadenylation
GV: germinal vesicle
GVBD: germinal vesicle breakdown
MCC: mitotic checkpoint complex
MMPs: matrix metallopeptidase.
